# Exploring the Role of Sprint Biomechanics in Hamstring Strain Injuries: A Current Opinion on Existing Concepts and Evidence

**DOI:** 10.1007/s40279-023-01925-x

**Published:** 2023-09-19

**Authors:** Christopher Bramah, Jurdan Mendiguchia, Thomas Dos’Santos, Jean-Benoȋt Morin

**Affiliations:** 1 School of Health and Society, University of Salford, Allerton Building, Frederick Road Campus, Salford, M6 6PU UK; 2 Manchester Institute of Health and Performance, Manchester, UK; 3 Department of Physical Therapy, ZENTRUM Rehab and Performance Centre, Barañain, Spain; 4 Department of Sport and Exercise Sciences, Manchester Metropolitan University, Manchester, UK; 5 Manchester Institute of Sport, Metropolitan University, Manchester, UK; 6 University Jean Monnet Saint-Etienne, Lyon 1, University Savoie Mont-Blanc, Inter-University Laboratory of Human Movement Biology, EA 7424, Saint-Etienne, France

## Abstract

Hamstring strain injuries are one of the most common injuries in sprint-based sports with the mechanism of injury considered the result of an interaction between applied mechanical strain and the capacity of the muscle to tolerate strain. To date, injury prevention and rehabilitation strategies have frequently focused on enhancing the capacity of the hamstrings to tolerate strain, with little consideration of factors directly influencing mechanical strain. Sprint running biomechanics are one factor proposed to influence the mechanical strain applied to the hamstrings that may be modified (towards reduced strain) within rehabilitation and injury prevention programs. This article aims to explore the theoretical mechanistic link between sprint running mechanics and hamstring strain injury, along with the available supporting evidence. In doing so, it hopes to provide practitioners with an understanding of mechanical parameters that may influence hamstring strain injury whilst also identifying areas for further research exploration.

## Key Points

**Table:** 

Sprint running mechanics are proposed to, and widely believed to influence mechanical strain placed on the hamstrings.
Several articles presented in this review provide evidence to support the mechanistic link between running mechanics and hamstring strain.
Targeting mechanical factors influencing hamstring strain may be beneficial in the prevention and rehabilitation of hamstring strain injuries and should be considered as an area of further study.

## Introduction

Hamstring strain injuries (HSIs) are one of the most common injuries affecting sprint-based sports [1], accounting for up to 24% of all injuries in football [2], 17% in track and field [3] and 22% in rugby union [4]. Within elite football, HSIs have been reported to have a significant performance and economic burden [5], leading to an average of 90 days and 15 games lost per club per season [6]. Of all HSIs, more than 47% are reported to occur during sprint running [7], with recurrence rates of 18% [2].

Hamstring injury prevention and rehabilitation strategies have currently focused on the development of eccentric strength qualities [8–11]. The premise behind this approach is that eccentric training develops tissue architecture and material properties, enhancing the capacity of the hamstrings to withstand strain encountered during maximal velocity running. The use of eccentric training interventions such as the Nordic hamstring exercise has proven beneficial in reducing injury rates [12], especially amongst individuals who adhere to established protocols [13]. However, despite several decades of research, the significance of HSI continues to increase, with incidence rates rising by 6.7% annually and injury burden by 9% [2, 14].

The increase in hamstring injury rates is likely multifactorial. First, increases in match play demands and exposure to maximal velocity running place individuals at greater risk of sustaining HSIs [2, 15]. Although eccentric strength training interventions have proven effective in certain contexts, there is contradictory evidence that suggests they may not fully address the impact of increased sporting demands [16]. Several studies have failed to identify consistent and strong associations between eccentric strength and future HSI [17, 18] or reinjury [19]. A recent meta-analysis by Impellizzeri et al. [16] also concluded that the preventative effect of eccentric strength training as a sole preventative strategy for HSI remains uncertain. This suggests that current injury prevention and rehabilitation programs do not adequately address all factors influencing HSI and reinjury. Therefore, it is essential to explore and address additional factors to improve the efficacy of current injury prevention programs.

**Fig. 1:**
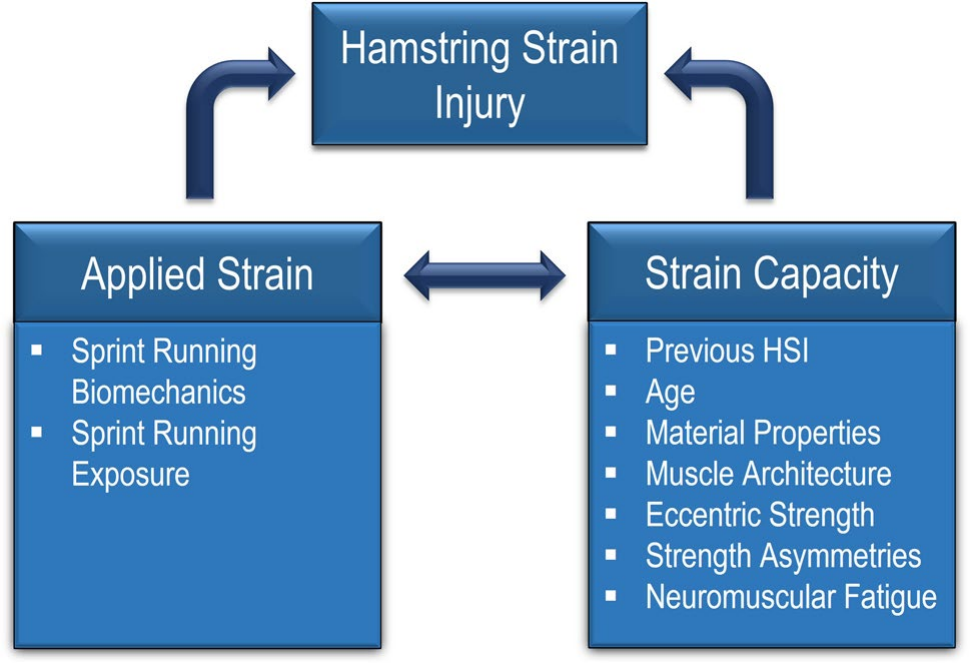
Biomechanical model of hamstring strain injury (HSI) highlighting the interaction between factors influencing applied strain and strain capacity

Within a biomechanical model of injury causation, muscle injury is considered the result of applied mechanical strain exceeding the tissue capacity to withstand strain [20] (Fig. 1). As such, a complex interaction between internal and external factors influencing tissue strain or strain capacity, is required for injury to occur [20]. Whilst eccentric strength training may provide some benefit in developing tissue capacity, it fails to acknowledge, or address, the role of applied mechanical strain.

Considering the majority of HSIs occur during sprint acceleration and high-to-maximal velocity running [7], it seems logical that biomechanics should be considered as a potential contributing factor influencing mechanical strain and thus injury development. Indeed, several expert opinion and qualitative studies suggest running mechanics should be considered within multimodal hamstring rehabilitation and injury mitigation programs [21–25], an approach adopted for other widespread musculoskeletal injuries such as anterior cruciate ligament [26–28] and athletic groin pain [29]. However, despite this, the injury mechanism rationale as to how sprint biomechanical parameters may influence tissue strain and HSI is scarcely discussed.

Therefore, this Current Opinion article aims to discuss the theoretical mechanistic link between sprint running mechanics and hamstring strain, along with the available supporting evidence. In doing so, this article aims to help clinical reasoning regarding biomechanical factors that could influence hamstring tissue strain and help clinicians identify potential biomechanical contributors to injury that could be targeted within the injury screening, mitigation and rehabilitation processes.

## Hamstring Biomechanics

Sprint acceleration and maximal velocity sprint running impose significant demands on the hamstrings. During acceleration, the hamstring muscle group generates large hip extensor torques contributing up to 15% of the total propulsive impulse [30]. Peak muscle forces range between 3 and 4.2 times body weight during stance and 8 times body weight during swing [30]. The biceps femoris muscle exhibits greater activation than the medial hamstrings during early stance [31], with the magnitude of activation during terminal swing contributing to the horizontal force produced during stance [32].

While the hamstrings undeniably play a crucial role in acceleration performance, the majority of the existing literature on injury causation focuses upon maximal velocity upright sprint running mechanics. As such, this article primarily focuses on maximal velocity sprint running mechanics and the potential injury implications.

**Fig. 2:**
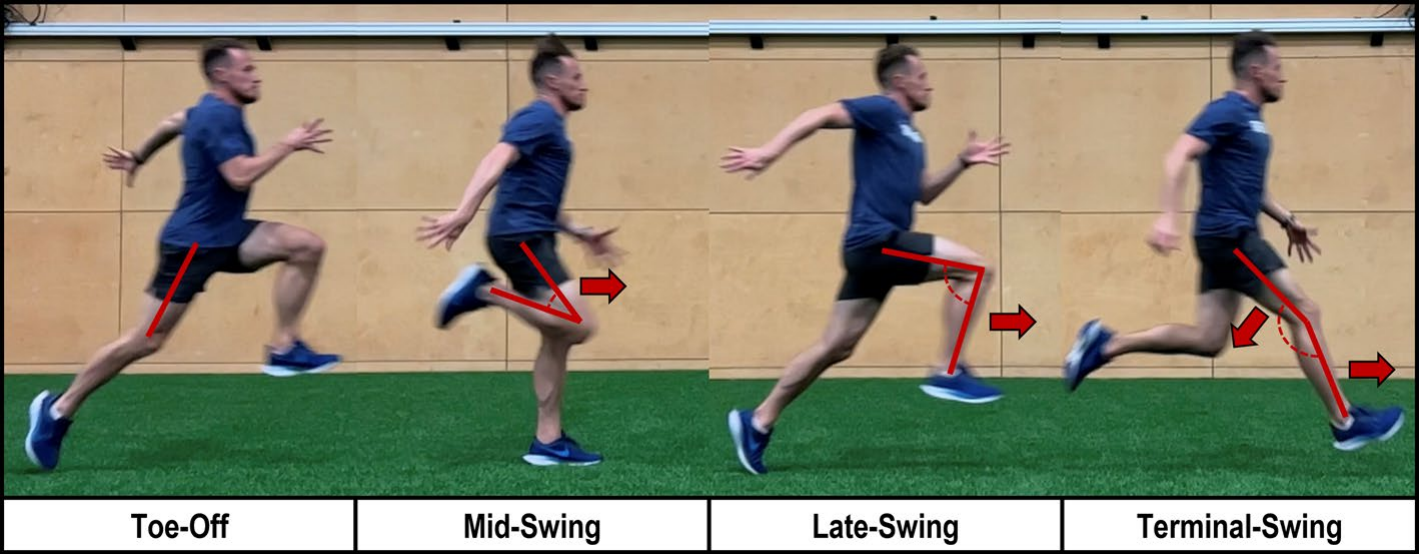
Pictorial representation of lower limb kinematics across phases of the sprint cycle

During high-to-maximal velocity sprint running, the swing and early stance phases are the most frequently studied regarding injury [33]. During the swing phase, rapid flexion and extension of the hip generates large angular accelerations of the shank, driving the knee into extension during the transition from late to terminal swing (Fig. 2). At terminal swing, peak muscle forces reach up to 10 times body weight [34]; with a range between 23.9 and 46.0 N/kg for the semimembranosus, 13.2–26.4 N/kg for the biceps femoris long head (BFLH), 10.4 N/kg for the short head, and up to 5.9 N/kg for the semitendinosus [35, 36]. Muscle–tendon lengths increase by approximately 10% for all hamstrings [36, 37], and peak muscle activity, lengthening velocities and negative work done by the hamstrings all occur during the transition between late and terminal swing [35–37].

Whilst the hamstrings must resist the motion-driven torque of the shank during swing, during stance they must resist joint torques generated by the vertical ground reaction force [34, 38]. Following ground contact, a sudden rise in vertical ground reaction force generates an external hip flexor and knee extensor moment that must be counteracted by the action of the hamstrings. An inability to counteract the external joint moments would likely drive the hip into further flexion and knee into extension, which could subsequently increase the active strain imposed on the hamstrings, influencing a tissue injury [38–40].

The high biomechanical demands of maximal velocity sprint running place the hamstrings at the upper limit of their physiological capacity. As both individual and team-sport demands continue to increase, with higher match-play running velocities and a greater frequency of exposures to sprint running [2, 15], the risk of injury will also increase. Greater running speeds subject the hamstrings to higher muscle forces [37], excitation levels [41] and tissue strain [42], particularly at the BFLH, while more frequent exposures and/or fixture congestion increase the possibility of tissue fatigue and accumulation of micro-trauma.

Consequently, even subtle changes in factors influencing the applied mechanical strain or strain capacity (as depicted in Fig. 1) may be sufficient to result in injury development. Therefore, to mitigate this risk, injury prevention programs should consider methods that both enhance the strain capacity of the hamstrings and modify the applied mechanical strain.

## Applied Anatomy

Based on the functional anatomy and link between anatomical segments, kinematic parameters during sprint running have the potential to influence applied mechanical strain. The hamstrings are biarticular muscles spanning both the hip and knee joints with distinct roles at each. Proximally, they attach to the pelvis via the ischial tuberosity, where the BFLH and semitendinosus form a conjoined tendon [43, 44]. In contrast to the semimembranosus and semitendinosus, the BFLH possesses an additional attachment to the sacrotuberous ligament, directly connecting it to the sacroiliac joint (SIJ) [45]. This attachment suggests the BFLH may contribute to, and be influenced by, pelvis and SIJ stability [45–47]. Thus, alterations in pelvis and SIJ kinematics will likely impact the strain distribution through the BFLH.

Distally, the semimembranosus and semitendinosus attach to the medial tibia, merging with the medial collateral ligament, meniscus and pes anserine. The biceps femoris descends distally, forming the biceps femoris short head that inserts on the lateral aspect of the fibula head, with fibres blending with the lateral collateral ligament, iliotibial band and surrounding fascia [43].

This complex anatomy provides clear connections between the hamstrings and proximal segments (trunk and pelvis), as well as distal segments of the knee and lower limb. Therefore, the hamstrings not only serve dual roles extending the hip and flexing the knee, but likely contribute to rotational and translational stability at the knee. The direct connections between the BFLH and sacrotuberous ligament also highlight the BFLH’s role in pelvis and SIJ stability. Consequently, mechanics at both proximal and distal segments can impact hamstring function and the applied mechanical strain.

## Kinematic Parameters

Regarding the mechanical influences on HSI, coaches and practitioners have rated certain kinematic parameters as “highly important” for hamstring injury prevention [22]. Parameters include an overstride running pattern, anterior pelvic tilt, lumbo-pelvic control, lumbar extension, back kicking of the trailing leg and trunk forward lean [22]. However, the mechanism by which such parameters may influence hamstring strain has not previously been detailed.

### Lumbo-Pelvic Control

Lumbo-pelvic control refers to the ability to control postural positions of the lumbar spine and pelvis during dynamic activity [48] and is widely considered to play a role in HSI [22, 48–51] and other sports injuries [29, 52, 53]. Based on the anatomical connections between the BFLH, sacrotuberous ligament and the pelvis, altered lumbo-pelvic control is proposed to cause ineffective force transfer across the trunk and pelvis, increasing the strain placed upon the hamstrings [46, 48].

The mechanistic link between lumbo-pelvic control and hamstring strain is logical given the proximal insertion of hamstrings to the ischial tuberosity. As the pelvis acts as a functional lever between the trunk and lower limbs, muscle forces can create angular accelerations across multiple segments, influencing strain imparted on the muscles of the contralateral limb. This concept of dynamic coupling is supported by the modelling work of Chumanov et al. [35], who investigated the influence of muscles spanning the trunk and pelvis on BFLH stretch during running. Interestingly, the iliopsoas of the contralateral leg was observed to increase BFLH stretch by more than 25 mm. This is likely explained by the iliopsoas accelerating the pelvis into anterior rotation, lengthening the proximal attachment site of the lead leg hamstring.

In contrast, the gluteus maximus, adductor magnus, and internal and external oblique were all observed to reduce the stretch imposed on the BFLH owing to their ability to resist anterior rotational forces generated by the iliopsoas [35]. Consequently, altered coordination between limbs during running along with fluctuations in neuromuscular control (thus pelvis and lumbar spine positions) may induce variable hamstring strain patterns resulting in either microdamage and subsequent tissue fatigue failure, or excessive hamstring strain causing acute injury.

Experimental studies provide evidence to support associations between altered lumbo-pelvic control and HSI [54–58]. In two prospective studies, altered trunk and pelvis muscle activity was observed in individuals sustaining a HSI [56, 57]. Franettovich Smith et al. [57] found nine Australian Football players who went on to sustain a new HSI displayed higher gluteus medius activity, whilst Schuermans et al. [56] reported lower activity of the gluteus maximus, erector spinae, and internal and external oblique during the swing phase in subsequently injured soccer players. Furthermore, using an unanticipated perturbation task, Higashihara et al. [58] identified a delayed onset of gluteus maximus and erector spinae muscle activity amongst individuals with a history of HSI. Therefore, from a clinical perspective, consideration of factors influencing lumbo-pelvic control appears logical and warranted within injury rehabilitation and prevention programs.

#### Trunk Lateral Flexion and Rotation

Deficits in controlling trunk lateral flexion and rotation are kinematic features associated with lumbo-pelvic control proposed to influence HSI [22]. Anatomical studies suggest that excessive rotation or side flexion of the trunk can alter the length–tension relationships of trunk musculature, reducing the ability to stabilise the pelvis and SIJ [44]. This negatively affects force transfer between limbs and across the pelvis, potentially leading to an increase in hamstring strain [48, 59]. In support of this concept, several studies have reported trunk muscle activity to influence SIJ stiffness [47], with SIJ stiffness in turn influencing hamstring muscle torque [60, 61], potentially influencing injury susceptibility.

Prospective evidence further supports the association between altered trunk control and HSI [54, 55]. In two studies, individuals who later sustained HSIs demonstrated increased trunk side flexion towards the injured limb during late swing [54, 55]. Additionally, prior work from the same author group observed reductions in oblique muscle activation occurring at the same timepoint among individuals with HSIs [56]. Although not reported by the authors, excessive trunk side flexion may be influenced by altered trunk, and in particular, oblique muscle coordination patterns.

The direct attachments of the oblique musculature to both the spine and pelvis, along with the orientation of the fibres, enable the obliques to resist trunk lateral flexion and rotation while exerting compressive forces on the pelvis and SIJ, enhancing force closure and reducing anterior pelvic tilt [44, 48]. Greater pelvis stability provided by oblique muscle activity would likely reduce strain applied to the proximal hamstrings while also enhancing force production capabilities [60]. Indeed, the work of Chumanov et al. [35] highlights that oblique muscle contraction can reduce the length of the biceps femoris by more than 10 mm, and thus likely the strain applied to the BFLH. Therefore, based on current evidence, reduced trunk and/or lumbo-pelvic control may lead to altered hamstring strain and function, increasing the propensity to future HSI.

#### Anterior Pelvic Tilt

Anterior pelvic tilt (APT) is widely cited as a kinematic contributor to HSI [62–64] and consistently reported by coaches and therapists working with sprinting athletes [21, 22]. From a functional anatomy standpoint, APT causes a rotation of the ischial tuberosity in the posterior and superior direction, lengthening the hamstrings and increasing tissue strain [65]. As the proximal moment arm of the hamstrings increase with hip flexion [66], the hamstring muscles are more susceptible to tissue length changes induced by segment rotations [67]. Therefore, uncontrolled or increased APT during swing and/or early stance when the hip is flexed will likely increase proximal hamstring strain and thus the risk of HSI.

Three studies have investigated the role of APT in HSI [54, 55, 68]. Schuermans et al. [54] conducted a prospective cohort study of 60 amateur soccer players in which four players who sustained a first-time hamstring injury were found to have greater APT compared with non-injured players; this finding was supported by retrospective data from Daly et al. [68]. However, in contrast, prospective work from Kenneally-Dabrowski et al. [55] did not observe this role of APT in hamstring injuries, with no differences observed between elite rugby players who went on to sustain a HSI and those who did not.

Whilst empirical data supporting associations between APT and HSI appear somewhat mixed, it must be acknowledged that current evidence is limited in both study numbers and sample sizes. With only four participants sustaining a HSI in current prospective work [54, 55], these studies are likely underpowered to detect between-group differences.

In contrast, evidence points towards a mechanistic link between APT and hamstring strain. Utilising shear wave elastography to measure the passive tension of the hamstrings, Nakamura et al. [69] found APT to increase tension by 13% for the semitendinosus, 26% for the semimembranosus and 31.5% for the biceps femoris. Additionally, Nagano et al. [65] identified maximal biceps femoris muscle length to coincide with peak APT and further work has shown APT to result in length changes to the hamstrings [70]. Considering recent work has shown interventions to effectively reduce APT during dynamic activities including sprint running [25, 62], APT likely represents a modifiable mechanical factor that could influence HSI risk.

#### Lumbar Extension

Qualitative work suggests excessive lumbar extension is viewed as moderately important by coaches and practitioners in HSI development [22]. The contribution of the lumbar spine to HSI has also been proposed in case reports and editorial publications [49, 71]. It is theorised that subtle impingements or nerve root irritation at the lumbar spine could lead to altered motor neuron function, potentially increasing the susceptibility to HSIs [71].

However, currently, there is limited evidence to support a direct causal relationship between lumbar spine pathology and HSI. Whilst studies exist reporting associations between lumbar “dysfunction”, neural tension and HSI [72, 73], it is widely acknowledged that “abnormal” magnetic resonance imaging features are frequently found amongst asymptomatic individuals [74]. Consequently, a causal link between lumbar spine pathology and HSI cannot be confirmed.

Additionally, to our knowledge, there appear to be no biomechanical studies investigating lumbar kinematics in HSIs. Therefore, the link between lumbar spine kinematics and HSI appears at best theoretical. Instead, it is perhaps more plausible that the mechanistic link between the two may be the result of kinematic coupling between lumbar spine extension and APT [75].

### Back-Side Mechanics

**Fig. 3:**
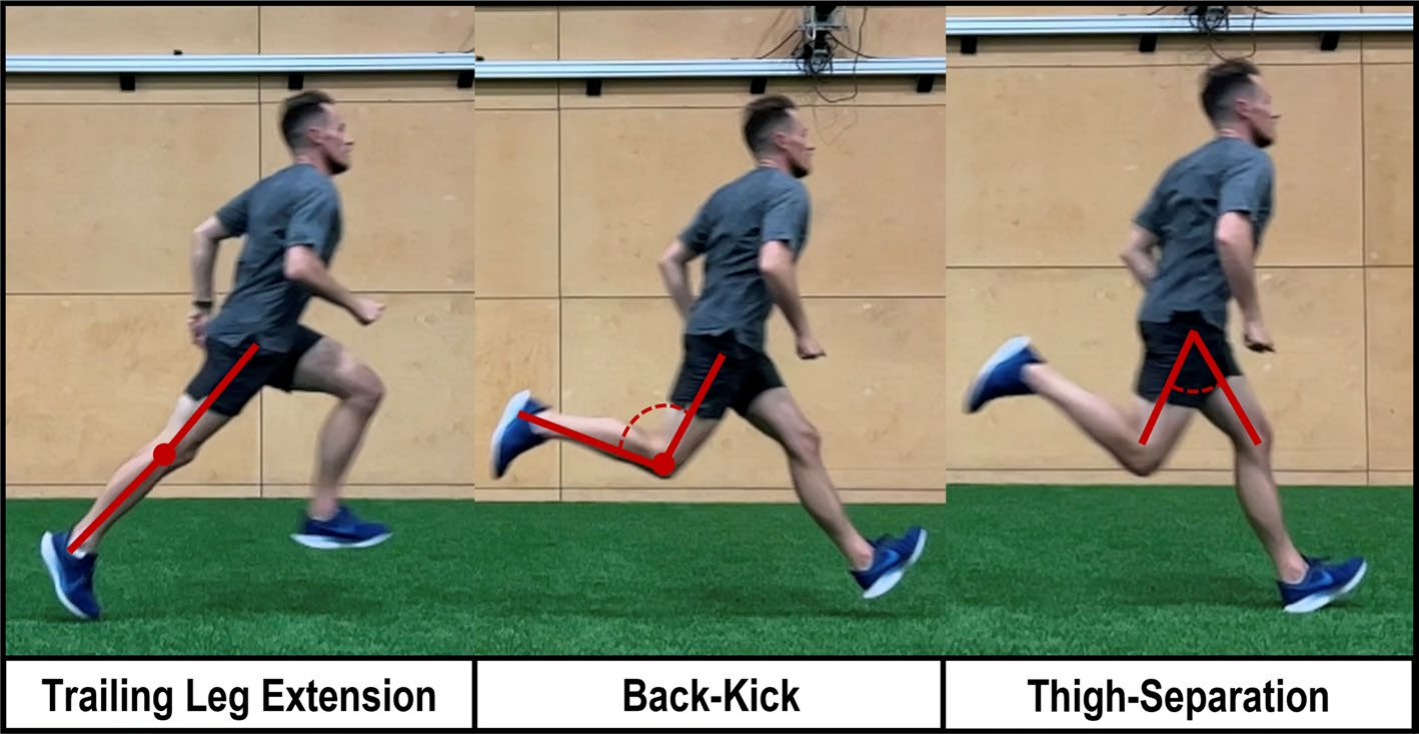
Pictorial representation of key features of back-side running mechanics

Back-side mechanics refers to the degree of lower-limb movements occurring behind the centre of mass during sprint running [76, 77]. This can appear as excessive extension of the trailing leg at toe off, “back-kicking” of the trailing leg during swing and large thigh separation angles (also termed inter-thigh angle) [78] (Fig. 3). Whilst minimising back-side and maximising front-side mechanics is reported to have implications for performance [79, 80], these parameters are also considered to have an important role in minimising hamstring strain [22, 76].

Based on the shared anatomical connections to the pelvis, back-side mechanics are thought to influence hamstring strain via increases in APT driven by length changes of the hip flexors [81]. Data from several studies seem to support this concept, with observations of maximal hip flexor length coinciding with peak APT and contralateral biceps femoris length [65, 81, 82]. As previously mentioned, the modelling work by Chumanov et al. [35] also highlights that iliopsoas muscle force of the trailing leg can significantly increase BFLH stretch [35], demonstrating the potential for back-side mechanics to influence APT and hamstring strain.

Two prospective studies have investigated associations between measures of back-side mechanics and HSI [78, 83]. Haugen et al. [78] investigated between-limb asymmetries in thigh-separation angles at touchdown in competitive sprinters, whilst Lahti et al. [83] utilised a novel measure of back-side running mechanics named the “kick-back” test as part of a multifactorial screening protocol in professional soccer players. However, neither study found differences between individuals sustaining a HSI and those remaining injury free.

Consequently, based on current evidence, there appear to be only theoretical associations between back-side mechanics and HSI. That said, limitations of the present experimental work must be acknowledged. First, few studies exist investigating the role of back-side mechanics in HSI populations and second, methods used to quantify back-side mechanics are inconsistent. The original work of Mann and Murphy [76] proposed back-side mechanics as a concept describing multiple kinematic features, including an extended trailing leg at toe off, greater duration of limb movements occurring behind the midline and large thigh separation angles at touchdown. Measuring isolated kinematic variables may not fully capture the degree of back-side mechanics in accordance with Mann and Murphy’s original concept [76]. Therefore, further work is required to establish how back-side mechanics is assessed and whether there is any association with HSI.

### Trailing Leg Extension

Trailing leg extension at toe off, often termed triple extension, is a mechanical feature commonly observed during acceleration. However, during maximal velocity sprint running, triple extension is considered a technical fault with the potential to influence hamstring strain and in turn HSI [22, 76]. As peak ground reaction forces are generated in the first half of the stance phase [84], triple extension is thought to be indicative of ineffective force production strategies [76, 79],﻿ where the athlete continues to push the ground after the point of peak ground reaction force occurrence. This may lead to several secondary mechanical consequences; including longer stance and shorter flight times, reducing the time available to reposition the lower limb for subsequent foot contact [76] increasing the need for faster limb switching. This has been proposed to lead to an over-stride gait pattern and/or an increase in forward trunk lean and “back-side” mechanics [76, 79].

Interestingly, Yu et al. [85] proposed increased leg extension at toe off may directly influence HSI. Investigating muscle–tendon kinematics during sprinting, they identified an additional point of hamstring strain occurring between mid to late stance and theorised that rapid knee extension at late stance may increase hamstring tissue strains, potentially influencing injury risk [85].

It is worth noting that no experimental studies have investigated the link between extension at toe off and HSIs. Current evidence against triple extension during maximum velocity running is based on associations with sprint performance only and, as such, the influence on HSI is perhaps due to secondary kinematic consequences, rather than triple extension per se.

### Maximum Hip Flexion Angle

Maximum hip flexion angle (MHF) is defined as the angle between the trunk and the thigh at late swing [79], considered a technical quality for optimising forward “projection” and sprint performance [76], and differentiates sprinters from middle-distance runners [86]. Greater MHF increases the height of the thigh allowing for a greater range of movement for the lower limb to unfold during swing and thus, generate large angular accelerations of the lower limb prior to ground contact [79, 87]. This, in turn, facilitates the production of a large ground reaction force during early stance [87], a key determinant of sprint running performance [84]. The work of both Clark et al. [87] and Sides [79] supports this concept with faster sprinters observed to have greater maximum hip flexion angles, smaller hip extension angles, and higher thigh extension and flexion velocities during both stance and swing.

In relation to HSIs, only three retrospective studies have reported MHF in individuals with HSI and have conflicting findings. Higashihara et al. [88] and Lee et al. [89] reported lower MHF in the previously injured limb, whilst Daly et al. [68] reported greater MHF. Interestingly, Higashihara et al. [88] reported concurrent findings of reduced biceps femoris EMG activity, greater knee flexion during terminal swing and reduced biceps femoris length, while Lee et al. [89] identified both reduced eccentric hamstring peak torque and angle of peak torque. Based on these additional findings, the author suggested reductions in MHF angle may represent post-injury kinematic adaptations acting to protect the hamstrings by reducing tissue strains associated with high MHF angles, compensating for reductions in eccentric hamstring strength capabilities.

Consequently, MHF is a biomechanical parameter that perhaps represents a performance-injury paradox. Whilst higher hip flexion angles appear to facilitate greater thigh extension velocities and ground reaction force generation, this would conceivably increase hamstring muscle forces and tissue strain during terminal swing.

### Over-Striding

Over-striding is characterised by the foot contacting the ground in front of the centre of mass. Kinematically, this may appear as increased hip flexion at contact, an extended knee, high tibial and foot inclination angles, and/or a lack of thigh and leg “retraction” during the swing phase [87, 90].

There are several mechanisms by which over-striding may influence hamstring strain and strain injury. First, over-striding has been shown to influence peak braking forces and braking impulse [90, 91]. Considering the hamstring muscle group is one of the main contributors to horizonal acceleration of the centre of mass [30, 32], greater braking induced by over-striding may require greater hamstring muscle forces to reaccelerate the centre of mass during maximal-speed running. Repeated over several sprinting bouts, this could lead to earlier muscle fatigue, reducing the internal resistance to tissue strain and increasing the vulnerability to fatigue-related tissue failure [92].

Second, over-striding has been shown to increase external hip flexor moments during stance, with high hip flexion angles also increasing hamstring muscle length [93, 94]. All other things equal, the combination of the two will ultimately expose the hamstrings to high muscle forces whilst in an elongated position and therefore increase the strain applied to the hamstrings.

Currently, only limited investigations have reported biomechanical variables associated with over-stride mechanics in HSI populations. Lee et al. [89] found no difference in between-limb hip joint angles or moments during the stance phase of running, whilst data from Daly et al. [68] appear to show greater hip flexion angles at contact amongst elite hurlers with a history of HSI when compared with controls. However, as kinetic data were not included in their analysis, whether the kinematic patterns influenced tissue loading or strain can only be speculated.

Therefore, whilst the contribution of over-stride mechanics to hamstring tissue strain is plausible, there is a lack of data investigating the association between the two. Additionally, no study has provided a comprehensive report of stance phase mechanics in HSI populations and this is therefore a recommended avenue for further research.

### Forward Trunk Lean

Forward trunk lean during sprinting has a significant impact on hamstring tissue length and force demands by influencing both kinematics and kinetics. Higashihara et al. [94] observed a significant increase in hamstring length across the entire stance phase when increasing forward lean during maximal speed running. The greater tissue length appeared to result from concurrent increases in APT and hip flexion angles in forward leaning trials, elongating the proximal attachment site and increasing hamstring strain.

Regarding kinetic consequences, increasing trunk forward lean causes an anterior displacement of the centre of mass, increasing the distance between the hip joint centre and ground reaction force vector. This gives rise to an increase in the external hip flexor moment, driving the hip into further flexion and thereby increasing hamstring strain [95]. To counteract this effect, greater hip extensor muscle forces must be generated [95]. When repeated over several loading bouts, the increased demand on the hip extensors can lead to increased metabolic and mechanical fatigue of the hamstrings, as well as a gradual increase in tissue microtrauma that may result in injury development.

Furthermore, the anterior shift in the centre of mass may cause a compensatory over-stride required to balance the anterior–posterior distance between the centre of mass and foot contact position [96]. As mentioned in Sect. 4.5, these over-stride mechanics may ultimately lead to increased hamstring tissue length and subsequent tissue strain.

Whilst coaches, therapists and some authors have anecdotally reported trunk forward lean to be associated with HSI [22, 97], limited evidence exists supporting associations between the two. In a case study of one individual sustaining a hamstring injury during testing, Schache et al. [98] observed a 3.3° increase in trunk flexion at initial contact of the subsequently injured limb. This coincided with greater peak vertical ground reaction force and loading rate, a 14% increase in peak hip extensor moment and a 30% increase in peak positive hip powers, all being mechanical patterns that will likely increase both hamstring strain and strain rate.

A further study by Kerin et al. [99] examined mechanisms of acute HSI in rugby players using a two-dimensional video analysis. Of eight injuries sustained during sprinting, all were observed to be in a position of increased trunk flexion. Therefore, whilst it seems plausible that forward lean trunk lean may influence hamstring tissue strain, further studies are required to investigate the association with HSI development.

## Conclusions

**Fig. 4:**
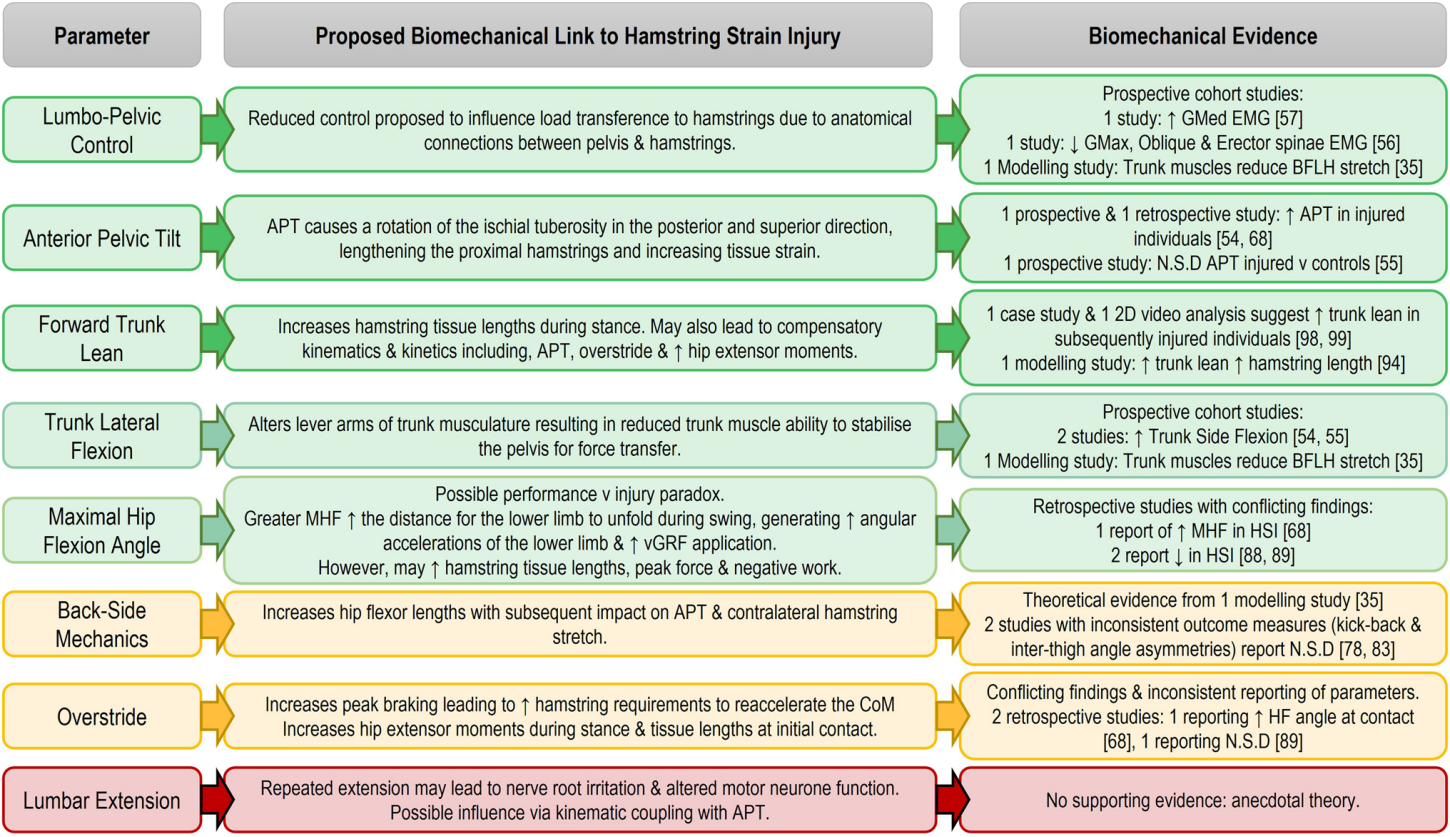
Graphical representation of current evidence detailing links between kinematics and hamstring strain injury. Green boxes indicate evidence from both experimental and modelling studies, yellow boxes indicate evidence from modelling studies only and red boxes indicate a theoretical association with hamstring strain injury with no experimental or modelling studies. ↑ increased, ↓ decreased, *APT* anterior pelvic tilt, *BFLH* biceps femoris long head, *CoM* centre of mass, *EMG* electromyography, *GMax* gluteus maximus, *GMed* gluteus medius, *HF* hip flexion, *HSI* hamstring strain injury, *MHF* maximal hip flexion, *n.s.d* non-significant difference, *vGRF* vertical ground reaction force

Current evidence from functional anatomy and biomechanical modelling studies appears to support the role of sprint running mechanics influencing hamstring tissue strain (Fig. 4). As strain is the primary mechanism of muscle injury, and several kinematic parameters appear to directly influence hamstring strain, it seems logical that kinematics could represent a modifiable risk factor for HSI. However, in current empirical studies, no isolated biomechanical parameter has been identified as a singular driver of hamstring strain. Rather, a combination of biomechanical parameters (i.e. movement quality or technical features) is frequently observed, suggesting that it is perhaps the interaction between multiple kinematic and kinetic features that influences the magnitude of applied strain leading to hamstring injury development.

The continued increase and evolution in the demands of both individual and team-sports competition, as well as periods of fixture congestion, represent a significant challenge to the prevention of HSI. As sporting demands increase, so do the biomechanical demands and applied strain on the hamstring muscle group, which will likely contribute to further increasing the incidence of HSIs. Consequently, successful injury prevention strategies require a more multifactorial and individualised approach, targeting both factors influencing tissue strain, such as mechanics and strain capacity.

However, the current lack of established in-field methods to assess sprint running biomechanics and definitive thresholds for defining “normal” or “abnormal” mechanics represents a barrier to the development of more detailed injury screening processes. Without such methods, the ability to reliably identify individuals who may benefit from targeted mechanical interventions remains limited and therefore should be an area of further research focus.
